# In Vitro Evaluation of 5-Fluorouridine as an Adjuvant to Antifungal Drugs and Molecular Insights into Resistance to This Compound in *Candida* Species

**DOI:** 10.3390/ijms27010171

**Published:** 2025-12-23

**Authors:** Monika Janeczko, Ewa Lenarczyk

**Affiliations:** 1 Department of Molecular Biology, Faculty of Medicine, The John Paul II Catholic University of Lublin, 20-708 Lublin, Poland; ewa.lena999@gmail.com; 2 Doctoral School of the John Paul II Catholic University of Lublin, 20-950 Lublin, Poland

**Keywords:** antifungals, *Candida*, efflux pump, 5-fluorouridine, FICI, synergism

## Abstract

This study evaluated the in vitro interaction of 5-fluorouridine (5-FUrd) with antifungal drugs and examined the role of efflux pumps in 5-FUrd resistance. Eleven reference *Candida* strains and twenty-three clinical *C. albicans* isolates from gynecological patients were tested. The antifungal activity of 5-FUrd alone and in combination with amphotericin B, fluconazole, voriconazole, caspofungin, and flucytosine was assessed using the checkerboard microdilution method. Efflux pump activity was evaluated using two inhibitors: carbonyl cyanide 3-chlorophenylhydrazone (CCCP) and verapamil. 5-FUrd exhibited antifungal activity against both the reference and clinical *Candida* strains, with MIC values ranging from 0.1 µg/mL to 409.6 µg/mL. The checkerboard assays revealed primarily no interactions in the reference *Candida* strains, whereas the reference *C. albicans* and clinical *C. albicans* isolates showed notable synergy between 5-FUrd and fluconazole, voriconazole, or caspofungin. The efflux pump inhibitors reduced the MICs of 5-FUrd in the resistant strains of *C. lusitaniae*, *C. kefyr*, and particularly *C. krusei*, suggesting efflux-mediated resistance mechanisms. This study highlights the potential of 5-FUrd, alone or combined with azoles or caspofungin, as an adjunct therapy against *Candida* infections. It also suggests that reduced susceptibility may be linked to efflux pump activity in certain strains.

## 1. Introduction

Over the past five decades, significant advancements in healthcare systems have markedly improved life expectancy and overall quality of life. However, these developments have coincided with a rising incidence of opportunistic infections, particularly among immunocompromised and hospitalized patients. One of the most concerning is systemic candidiasis, which remains a leading cause of infection-related morbidity and mortality worldwide [1]. Candidiasis is primarily a healthcare-associated infection caused by *Candida* species, which are typically commensal but can become pathogenic in certain conditions. Clinically, candidiasis may manifest in diverse forms, ranging from superficial infections, e.g., oral and vulvovaginal candidiasis, to deep-seated and life-threatening invasive candidiasis. More than 18 *Candida* species have been implicated in human infections, though six are responsible for over 95% of invasive cases. *Candida albicans* remains the most frequently isolated species, accounting for approximately 63–70% of cases [2,3]. Nevertheless, non-*albicans Candida* (NAC) species, such as *C. tropicalis*, *C. parapsilosis*, *C. krusei* (currently named *Pichia kudriavzevii*), *C. glabrata* (currently named *Nakaseomyces glabratus*), and *C. auris* (currently named *Candidozyma auris*), have emerged as equally relevant clinical pathogens. These species are commonly found in the environment, on the skin, or as part of the normal mucosal flora [4]. Although the majority of severe infections are caused by the predominant *Candida* species, emerging evidence highlights the pathogenic potential of less common species, such as *C. guilliermondii*, *C. kefyr*, *C. lusitaniae*, *C. lipolytica*, *C. norvegensis*, and *C. rugosa*. Despite their lower frequency, these species may cause invasive infections that are clinically challenging due to variable antifungal susceptibility profiles and diagnostic difficulties [5].

The current antifungal therapy relies on five main drug classes, polyenes, azoles, echinocandins, pyrimidine analogues, and allylamines, which target distinct components of the fungal cell, including the cell membrane, cell wall, and nucleic acid synthesis pathways [6]. While these agents have significantly improved clinical outcomes, the therapeutic paradigm is increasingly being challenged by the emergence and dissemination of antifungal resistance. The widespread and prolonged use of antifungal agents, particularly azoles and polyenes, in both clinical and agriculture settings has been linked to the rapid selection of resistant *Candida* strains. Resistance in *Candida* arises through a variety of molecular mechanisms, many of which can occur in parallel within the same isolate, contributing to multidrug resistance (MDR) and limiting treatment efficacy [7]. These include overexpression or mutation of drug targets, drug sequestration, enhancement of drug efflux, and reduction in drug uptake. Beyond these primary mechanisms, *C. albicans* demonstrates remarkable genomic and phenotypic plasticity. Chromosomal aneuploidy, involving the duplication or loss of specific chromosomes or chromosomal regions, can increase the copy number of genes associated with resistance. Phenotypic switching, which entails reversible changes in gene expression and cell morphology, also contributes to variations in antifungal susceptibility [3,7].

These overlapping resistance mechanisms underscore the adaptive capacity of *C. albicans* under antifungal pressure and highlight the urgent need for improved therapeutic strategies. A combination therapy represents a promising approach, offering several advantages over monotherapy: synergistic targeting of multiple cellular pathways, enhanced efficacy at lower drug concentrations, reduced host toxicity, and the potential to delay or prevent resistance development [8]. Synergy has been reported not only among antifungal agents but also between antifungals and non-antifungal compounds, including antibacterials, synthetic peptides, proton pump inhibitors, glucocorticoids, non-steroidal anti-inflammatory drugs, antiprotozoals, anti-rheumatic agents, calcineurin inhibitors, Hsp90 inhibitors, calcium homeostasis regulators, and various bioactive natural products and phytochemicals [6,9,10,11,12,13,14,15,16]. Clinically, the combination antifungal therapy has shown efficacy in difficult-to-treat infections, including central nervous system candidiasis and intra-abdominal abscesses, with combinations like amphotericin B (AMB), with flucytosine (5-FC) serving as a standard of care [17].

Given the growing interest in nucleoside analogs as potential modulators of antifungal activity, we recently explored the therapeutic properties of 5-fluorouridine (5-FUrd). Our previous studies demonstrated that 5-FUrd exhibits broad antifungal activity across several *Candida* species. Among the tested species, *C. albicans* and *C. parapsilosis* showed the highest sensitivity to 5-FUrd, correlating with inhibition of multiple virulence-associated processes, including biofilm formation, adhesion, morphogenesis, proteinase secretion, and hemolytic activity [18]. 5-FUrd is a fluoropyrimidine nucleoside analog, a cell-permeable RNA precursor, and a prodrug of 5-fluorouracil (5-FU). Importantly, it is metabolically activated through the uridine permease/uridine kinase pathway, allowing it to bypass resistance mechanisms associated with 5-FC and certain azole drugs. 5-FUrd inhibits *C. albicans* growth by targeting thymidylate kinase (CaTMPK), a key enzyme in dTTP biosynthesis. CaTMPK phosphorylates dTMP to dTDP, which is subsequently converted to dTTP by nucleoside diphosphate kinase (NDPK). Its fungal selectivity is supported by structural differences from human thymidylate kinase (hTMPK), notably the Ca-loop, a unique surface-exposed catalytic element, whose deletion impairs *C. albicans* growth, as well as by its 15-fold higher catalytic efficiency compared to hTMPK. CaTMPK also efficiently converts dUMP to dUDP and 5-FdUMP to 5-FdUDP, enhancing 5-FUrd cytotoxicity. These findings highlight the dual antifungal and antivirulence properties of 5-FUrd and support its evaluation as a candidate for combination therapy [19].

Building on these observations, the present study investigates 5-FUrd activity in combination with standard antifungal agents against reference *Candida* strains and clinical isolates of *C. albicans*. Because some strains showed reduced susceptibility in our previous assays, we also evaluated whether efflux pump activity contributes to decreased sensitivity. Understanding these mechanisms is essential for determining the therapeutic potential of 5-FUrd and identifying species or strain backgrounds most likely to benefit from combination treatments.

## 2. Results and Discussion

In recent years, combination therapy has received growing interest as a strategy to enhance the efficacy of antifungal treatments, particularly in infections caused by *Candida albicans*, including drug-resistant strains. Such combinations can be broadly categorized based on their mechanisms of action. The main groups include: (i) antifungal agents combined with efflux pump inhibitors, (ii) agents that reverse resistance-related efflux to enhance intracellular drug accumulation, and (iii) compounds that disrupt cell wall or cell membrane integrity to improve drug permeability. The interaction between antifungal drugs depends on the selected method, the antifungal combination, the sequence of administration, and the genus, species, and strain of the pathogen [10,20].

In this study, the in vitro antifungal activity of 5-fluorouridine (5-FUrd) was assessed both as a monotherapy and in combination with five commonly used antifungal agents: amphotericin B (AMB), fluconazole (FLU), voriconazole (VOR), caspofungin (CAS), and flucytosine (5-FC). The tests were conducted against eleven reference *Candida* strains and 23 clinical isolates of *C. albicans*.

We included the major clinically relevant *Candida* species, reflecting their epidemiological distribution in Europe and the United States (USA), where *C. albicans* accounts for approximately 50% of infections, followed by *C. glabrata* (30%), *C. parapsilosis* (12%), *C. tropicalis* (7%), and *C. krusei* (1%) [21]. *C. albicans* remains the most prevalent species among hospitalized and immunocompromised patients, with a growing trend toward azole resistance. Non-albicans *Candida* species are increasingly observed in specific high-risk populations and exhibit diverse antifungal susceptibility profiles, including reduced susceptibility to azoles and echinocandins [22,23]. 5-FUrd exhibited inhibitory activity against these species, with MIC values ranging from 0.2 µg/mL to 51.2 µg/mL. *C. auris* also represents a pathogen of major clinical significance, causing severe healthcare-associated infections worldwide and exhibiting a high capacity for skin colonization, environmental persistence, and patient-to-patient transmission. It is characterized by multidrug resistance, immune evasion, and high mortality rates, which has led to its classification by the World Health Organization (WHO) as a critical fungal pathogen [24,25]. Our study demonstrated that 5-FUrd exhibited weak inhibitory activity against *C. auris*, with MIC value of 409.6 µg/mL. Less common species, including *C. lusitaniae*, *C. kefyr*, and *C. norvegensis*, which were also examined in the present study, occur predominantly in severely immunocompromised patients and may exhibit distinct antifungal resistance profiles, including resistance to AMB or FLU [23,26]. The MIC values of 5-FUrd for these three species ranged from 25.6 to 51.2 µg/mL. Additionally, because *Candida* spp. can also cause disseminated bloodstream infections (candidemia) and the infection of tissues or deep organs (candidiasis) [27], two *C. albicans* blood isolates (ATCC 90028 and ATCC 90029) were included for testing. A significant difference in susceptibility to 5-FUrd was observed between these isolates, with MICs of 0.8 µg/mL and 204.8 µg/mL for *C. albicans* ATCC 90028 and *C. albicans* ATCC 90029, respectively.

Table 1 summarizes the MIC values of 5-FUrd and the reference antifungal agents for the tested reference *Candida* species.

Drug interactions and corresponding Fractional Inhibitory Concentration Index (FICI) values were assessed using the checkerboard assay. Despite its clinical significance, *C. auris* was not included in these experiments, as we demonstrated markedly reduced susceptibility of this species to 5-FUrd compared with other *Candida* species tested. Similarly, the *C. albicans* ATCC 90029, which also exhibited a high MIC value for 5-FUrd, was excluded from drug interaction studies and from the evaluation of potential adjuvant activity. Consequently, *Candida* strains exhibiting a more pronounced response to this compound were prioritized to allow a clearer assessment of potential synergistic interactions. As shown in Table 2, combinations of 5-FUrd with the tested antifungal agents predominantly exhibited indifferent effects against all *Candida* species. Notably, synergistic interactions were observed between 5-FUrd and FLU as well as VOR against *C. albicans* ATCC 10231, and between 5-FUrd and CAS against *C. albicans* ATCC 10231, *C. kefyr*, and *C. norvegensis*. In addition, synergistic effects between 5-FUrd and FLU or VOR were also detected for *C. albicans* ATCC 90028, a strain isolated from a systemic infection. These findings highlight the need for cautious interpretation of specific drug combinations in a species- and strain-dependent context.

The antifungal activity of 5-FUrd and antifungal drugs was further evaluated against 23 clinical *C. albicans* isolates. The MIC values of 5-FUrd ranged from 0.1 µg/mL to 12.8 µg/mL. Interestingly, 5-FUrd was effective against *C. albicans* isolates that were resistant to antifungal drugs (Table 3).

**Figure 1:**
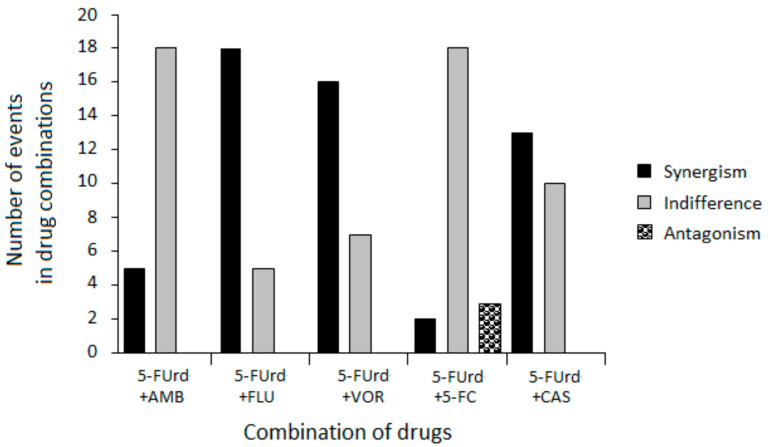
Summary of antifungal interactions between 5-FUrd and standard antifungal agents across *C. albicans* clinical isolates.

Based on the FICI values, the interactions among the drug combinations ranged from synergistic to antagonistic, depending on the specific combination and strain. However, several consistent patterns emerged, as shown in Table 4 and Figure 1.

The combination of 5-FUrd with AMB generally resulted in indifferent effects, with occasional synergy. Importantly, no antagonistic interactions were detected for this combination, suggesting its potential safety for combined use.

The most notable findings in this study were the predominantly synergistic interactions observed between 5-FUrd and the FLU and VOR azoles against the reference and isolates of *C. albicans*. Similarly, the combination of 5-FUrd with CAS also exhibited primarily synergistic activity. Comparable synergistic effects have been previously reported for another nucleoside analogue, ribavirin, which demonstrated synergistic activity when combined with such azoles as FLU, itraconazole (ITZ), and posaconazole (PSZ) [28,29]. Moreover, ribavirin in combination with CAS has shown a synergistic anti-*C. albicans* effect by inhibiting planktonic growth, disrupting preformed biofilms, and impairing hyphal development. This combination also enhances in vivo efficacy against resistant strains and promotes fungal cell death associated with metacaspase activation [30]. These results suggest that the rationale for combination therapy is to maximize antifungal effects by simultaneously targeting distinct fungal pathways to produce synergistic effects. Such synergy has been demonstrated for the combination of flucytosine (5-FC), i.e., a nucleoside analogue of cytosine, with FLC, VOR, or PSZ, particularly against *Cryptococcus neoformans* and *C. glabrata* [31,32,33]. The underlying mechanism of these fungistatic interactions may involve increased cellular uptake of 5-FC due to azole-induced disruption of ergosterol biosynthesis and consequent alterations in membrane permeability. Moreover, synergy between 5-FC and CAS has been demonstrated against *A. fumigatus* and *C. neoformans*. In this case, the disruption of the fungal cell wall facilitated the action of 5-FC by increasing its intracellular concentration [32,34,35]. A similar mechanism may account for the interaction between 5-FUrd and azoles or CAS observed in our study.

Finally, the interaction between 5-FUrd and 5-FC was mainly indifferent with a single instance of antagonism observed. This lack of synergy is likely due to their shared mechanism of action. Both drugs are metabolized into 5-FU derivatives that interfere with nucleic acid synthesis [19,31]. The competition for the same metabolic activation routes may result in pathway saturation, limiting the additive benefit of combining these two agents. As a consequence, no enhancement of antifungal activity was observed beyond what was achieved by either drug alone. Similar observations were reported for the combination of ribavirin and 5-FC, which did not show synergistic activity against any of the tested *Candida* strains [28]. However, some combinations offer good results when using similar or identical pathways. For example, the combination of terbinafine (TRB) with broad-spectrum triazoles, such as ITZ, VOR, ravuconazole (RVC), or albaconazole, has shown synergistic activity against *A. fumigatus*, *Scedosporium prolificans*, dimorphic fungi such as *Sporothrix schenckii*, and opportunistic molds, including *S. brevicaulis*, *Fusarium*, and *Paecilomyces*, as well as dematiaceous fungi and yeasts like *C. glabrata* [20,36,37,38,39,40]. TRB and azoles are ergosterol biosynthesis inhibitors, but TRB acts on different targets than azoles; specifically, it inhibits squalene epoxidase, whereas azoles inhibit lanosterol 14α-demethylase [41].

The promising synergistic activity of 5-FUrd with FLU, VOR, and CAS, particularly against resistant or partially resistant *C. albicans* isolates, suggests potential for repositioning 5-FUrd as part of novel combination regimens. However, the variability in interaction profiles across *Candida* species and strains emphasizes the importance of individualized susceptibility testing.

Nevertheless, a critical challenge in translating 5-FUrd into clinical antifungal applications is its potential cytotoxicity toward human cells. 5-FUrd is not pharmacologically active per se but exerts its effects following intracellular enzymatic conversion to 5-FU. Unlike the extensively characterized 5-FU and related analogues, systematic IC_50_/EC_50_ data for 5-FUrd in normal human cell lines are limited.

5-FU, a widely used antimetabolite in chemotherapy, exerts its antiproliferative effects primarily through inhibition of thymidylate synthase and incorporation into RNA and DNA, resulting in impaired nucleic acid synthesis and induction of cell death in proliferating cells. Previous studies in colorectal carcinoma cells (HT-29) demonstrated that 5-FU interferes with pre-rRNA processing pathways, contributing to its cytotoxic impact on tumor cell viability in vitro [42]. However, cytotoxicity of 5-FU is not strictly limited to malignant cells. When administered in vitro without delivery modifiers, 5-FU exhibits substantial toxicity toward both cancerous and normal cells. For example, in a comparative analysis of pure 5-FU on normal colon fibroblastic cell lines (CCD112) and colorectal cancer cells (HCT116), 5-FU alone effectively reduced viability in both cell types at micromolar concentrations after 72 h of treatment, indicating limited intrinsic selectivity of the unmodified compound. Such findings are consistent with reports that pure 5-FU causes significant cell death across a variety of nasopharyngeal normal and cancer cell lines, underscoring potential drawbacks of administering unformulated 5-FU in the absence of targeted delivery systems [43]. The propensity of 5-FU to induce cytotoxicity in non-malignant cells extends beyond epithelial cell models. In cardiomyocyte and colon cancer cell co-culture systems, 5-FU induced IC_50_ at substantially higher concentrations in rat cardiocytes (400 µM) compared to human HT-29 colon carcinoma cells (4 µM), suggesting a differential dose–response dynamic between healthy and malignant cell types, although higher doses still triggered apoptosis in cardiomyocytes [44]. Additionally, 5-FU has been shown to induce endothelial and cardiomyocyte stress responses, including reactive oxygen species (ROS) generation and features of cellular senescence, further implicating potential toxicity to non-target cell populations and highlighting mechanisms that may underlie cardiovascular side effects observed clinically [45]. Conversely, certain normal cell types demonstrate relative resilience to 5-FU at clinically relevant concentrations. For example, in vitro studies on cultured human corneal epithelial cells and keratocytes did not observe significant cytotoxic effects at 5-FU concentrations below 1% over short exposure periods of up to two hours, although higher doses and longer incubation times produced measurable inhibition of migration and viability [46]. These differences between cell types emphasize the importance of experimental conditions when interpreting cytotoxicity profiles. Collectively, these data suggest that while 5-FU is an effective antiproliferative agent against cancer cells, its selectivity in vitro is imperfect, with notable cytotoxic effects on certain normal cell lines at higher concentrations.

Similarly, available results show that 5-FUrd potently inhibits proliferation of tumor-derived mammalian cells, primarily through RNA-directed mechanisms, in addition to DNA-related effects. Previous investigations indicated that 5-FUrd interferes with RNA processing. In Sarcoma-180 murine tumor cells, micromolar concentrations of 5-FUrd induced dose-dependent alterations in small nuclear RNA (snRNA) structure and turnover. At 10 µM (2.62 µg/mL), changes in the electrophoretic mobility of U4 and U6 snRNAs were observed under non-denaturing conditions, while U1 snRNA turnover was selectively reduced, exceeding control levels by over 100% at 48 h. These disruptions in RNA splicing likely impair global mRNA maturation and protein synthesis, providing a basis for the prolonged growth inhibition seen after brief exposure [47]. The irreversible cytotoxicity of 5-FUrd was demonstrated in murine lymphoma L5178Y cells. Even 1-hour exposure to 1.5 µM (0.39 µg/mL) caused sustained proliferation arrest for at least a week. In contrast, 5-fluoro-2′-deoxyuridine (5-FdUrd) required continuous exposure to inhibit proliferation, and short-term exposure to high concentrations (1000 µM corresponding to 246 µg/mL) produced only reversible effects, highlighting the unique RNA-directed, irreversible action of 5-FUrd [48]. Additional evidence comes from K-562 erythroleukemia cells, where incorporation of 5-FUrd into RNA polymerase II transcripts was reduced using α-amanitin (1–5 µg/mL). This treatment decreased the incorporation of 5-FUrd into poly(A)+ RNA by up to 60% and antagonized its growth-inhibitory effects, directly linking cytotoxicity to mRNA incorporation and transcriptional disruption [49]. Taken together, these results indicate that 5-FUrd exhibits biological activity already at low micromolar concentrations (approximately 1–10 µM, corresponding to 0.4–2.6 µg/mL), inducing antiproliferative effects in tumor-derived mammalian cell lines. In comparison, another structurally related nucleoside, 5′-deoxy-5-fluorouridine (5′-DFUR), exhibits cytotoxic activity in tumor cell lines at micromolar concentrations, while demonstrating markedly reduced toxicity toward normal human bone marrow stem cells (LD_50_ = 580 µM). Importantly, 5′-DFUR is a pharmacologically inactive prodrug that requires intracellular conversion to 5-FU by thymidine phosphorylase. Consequently, its cytotoxic effect is strongly dependent on tissue- and cell-type-specific expression of this enzyme, which complicates direct quantitative comparison of its activity with that of 5-FUrd [50].

Despite pronounced cytotoxic effects observed in tumor cells, the impact of 5-FUrd on normal cells and whole-organism models remained insufficiently characterized. To address this gap, in our previous study we evaluated the toxicity of 5-FUrd in zebrafish (*Danio rerio*) embryos and human erythrocytes at concentrations ranging from 0.4 to 8 µg/mL (1.5–30.5 µM). These values were selected based on the MICs of two FUrd-sensitive species, *C. albicans* (MIC 0.4 µg/mL) and *C. parapsilosis* (MIC 0.2 µg/mL). Zebrafish embryos exposed for 96 h post-fertilization exhibited no significant differences in mortality (≤14%), heart rate, or gross morphology compared with control groups. Likewise, human erythrocytes showed less than 10% hemolysis across all tested concentrations, indicating minimal cytolytic activity [18].

Collectively, these results indicate a differential cytotoxicity profile of 5-FUrd: while the compound potently inhibits rapidly proliferating tumor cells at submicromolar to low micromolar concentrations, it exerts minimal toxicity toward non-dividing cells, as well as in a whole-organism model, at concentrations up to 8 µg/mL. This apparent selectivity is consistent with its RNA-directed mechanism of action and, to some extent, mirrors the therapeutic window reported for 5-FU in cancer therapy.

In summary, the integration of classical cell-based studies with our zebrafish and erythrocyte data indicates that 5-FUrd possesses a narrow but potentially exploitable therapeutic window. While its potent RNA-mediated cytotoxicity necessitates caution in proliferative host tissues, the low toxicity observed in non-dividing cells and whole-organism models at antimicrobial concentrations supports further evaluation of 5-FUrd as an antifungal agent against selected *Candida* species, provided that dosing and exposure are carefully controlled. 

In our study some *Candida* strains were less susceptible to 5-FUrd (MIC ≥ 6.4 µg/mL), including *C. albicans* ATCC 90029, *C. auris*, *C. glabrata*, *C. krusei*, *C. tropicalis*, *C. lusitaniae*, *C. kefyr*, *C. norvegensis*, and four clinical strains of *C. albicans*. Therefore, we checked the efflux pump activity using the efflux pump inhibitors (EPIs), the nonspecific—carbonyl cyanide 3-chlorophenylhydrazone (CCCP) and the specific calcium channel blocker—verapamil for the 5-FUrd-resistant strains [51,52]. Indeed, efflux pump-mediated resistance has been well-documented in *Candida* species and includes ATP-binding cassette (ABC) transporters, such as CDR1 and CDR2, as well as major facilitator superfamily (MFS) pumps like MDR1 [53].

As shown in Table 5, significant changes were reported in the 5-FUrd MICs for *C. krusei* after the EPI treatment. Decreases in the MIC values were also observed for *C. lusitaniae* and *C. kefyr* upon the exposure to 10 µg/mL of CCCP or verapamil, respectively. These results may indicate the dysfunctional transport of 5-FUrd in these *Candida* species. No differences were found in the 5-FUrd MICs for the other tested strains. These results suggest a possibility of the existence of another specific pathway that could induce the development of 5-FUrd resistance in these *Candida* species.

## 3. Materials and Methods

### 3.1. Microorganisms and Compounds

The antifungal activity of 5-fluorouridine (5-FUrd) was evaluated in vitro in comparison and in combination with standard antifungal agents: amphotericin B (AMB), fluconazole (FLU), voriconazole (VOR), caspofungin (CAS), and flucytosine (5-FC). Reference strains of *Candida* were obtained from the American Type Culture Collection (ATCC): *C. albicans* ATCC 10231, *C. albicans* ATCC 90028, *C. albicans* ATCC 90029, *C. auris* ATCC MYA-5001, *C. glabrata* ATCC 15126, *C. krusei* ATCC 14243, *C. parapsilosis* ATCC 22099, *C. tropicalis* ATCC 13803, *C. lusitaniae* ATCC 34449, and *C. kefyr* ATCC 204093. In addition, 23 clinical *Candida* strains were isolated from the reproductive tracts of patients treated at the Jan Boży Independent Public Provincial Hospital in Lublin, Poland. Clinical isolates were identified using the VITEK 2 YST IC CARDS (bioMérieux, Warsaw, Poland). All strains were routinely cultured in YPD broth (1% yeast extract, 2% peptone, 2% dextrose) at 37 °C in a shaking incubator (200 rpm) for 24 h. Sabouraud Dextrose Agar (Biocorp, Warsaw, Poland) and RPMI-1640 medium with L-glutamine and without sodium bicarbonate (Sigma Aldrich, St. Louis, MO, USA) were used as well. The tested drugs, dimethyl sulfoxide (DMSO), verapamil, carbonyl cyanide 3-chlorophenylhydrazone (CCCP), and other reagents were purchased from Sigma Aldrich (St. Louis, MO, USA).

### 3.2. Antifungal Susceptibility Testing

The Minimum Inhibitory Concentrations (MICs) of 5-FUrd and antifungal drugs were determined with the broth dilution method according to the Clinical and Laboratory Standards Institute (CLSI) [54] and as previously described by Khabnadideh et al. [55]. Three colonies of the microorganism were transferred to 5 mL of sterile 0.85% NaCl, and turbidity was adjusted to 0.5 McFarland. The inoculum was then diluted 1:1000 in RPMI-1640 medium. Two-fold serial dilutions of each tested agent were prepared using microtiter plates. Subsequently, 100 µL of inoculum was added to each well, giving a final volume of 200 µL per well. The plates were incubated at 37 °C for 48 h. Wells containing uninoculated medium served as sterility controls. The MIC was defined as the lowest concentration of the compound that completely inhibited visible growth. All experiments were performed in triplicate.

### 3.3. Determination of Fractional Inhibitory Concentration Index (FICI)

The interactions between 5-FUrd and antifungal drugs against reference *Candida* species and *C. albicans* clinical isolates were determined using the chequerboard microdilution method and included determinations of the MIC of each drug alone. Two-fold serial dilutions of drugs were used for MIC determinations. The first antifungal of the combination was serially diluted along the ordinate, while the second drug was diluted along the abscissa. The resulting chequerboard contained each combination of two antifungals, with tubes that contained the highest concentration of each antifungal at the opposite corners. The plates were incubated at 37 °C for and the MIC end points were read after 48 h. The MIC value was determined as the lowest concentration of the drugs (alone or in combination) that inhibited growth by 100%, compared with that of the drug-free wells. The interaction was classified on the basis of the fractional inhibitory concentration index (FICI). The FICI was calculated using the formula: FICI = FIC_A_ + FIC_B_ = (MIC_AB_/MIC_A_) + (MIC_BA_/MIC_B_), where MIC_AB_ is the minimum inhibitory concentration (MIC) of drug A (5-FUrd) tested in combination, MIC_A_ is the MIC of drug A tested alone, MIC_BA_ is the MIC of drug B (the other antifungal drug) tested in combination, and MIC_B_ is the MIC of drug B tested alone. Synergy was defined as a FICI ≤ 0.5, indifference as a FICI between >0.5 and ≤4, and antagonism as a FICI > 4 [56]. All experiments were performed in triplicate.

### 3.4. Efflux Pump Activity

To investigate the role of efflux pump activity in resistance to 5-FUrd, selected *Candida* strains showing reduced susceptibility were tested, including *C. albicans* ATCC 90029, *C. auris*, *C. glabrata*, *C. krusei*, *C. tropicalis*, *C. lusitaniae*, *C. kefyr*, *C. norvegensis*, and four *C. albicans* clinical isolates (no. 10, 12, 17, and 23). Efflux pump inhibitors (EPIs), CCCP and verapamil, were tested at two final concentrations (5 µg/mL and 10 µg/mL). To ensure that these concentrations did not independently affect fungal growth, control wells with the inhibitors alone were included. The MICs of 5-FUrd were determined before and after the addition of EPIs to the RPMI-1640 medium. A reduction in MIC upon the addition of the inhibitors was interpreted as evidence of efflux pump involvement in the resistance mechanism [52,53].

## 4. Conclusions

In conclusion, our findings highlight the antifungal potential of 5-FUrd in combination therapy, especially together with azoles, FLU and VOR, and CAS. Future studies should be extended to include additional *Candida* strains, especially clinical isolates and other antifungal drugs, and to investigate the in vivo efficacy of 5-FUrd-based combinations, with particular emphasis on their role in overcoming resistance in clinical *Candida* infections. Moreover, further research should focus on elucidating the molecular mechanisms underlying the observed interactions between 5-FUrd and the tested antifungal agents to better understand their pharmacodynamic profiles and optimize therapeutic strategies. Importantly, our results also indicate that resistance to 5-FUrd can emerge in certain *Candida* strains through active efflux mechanisms, which may represent a potential limitation to its therapeutic application. Finally, it should be noted that in vivo studies to date have been limited to animal models (zebrafish) and non-proliferating human cells (erythrocytes), and that the complete toxicity profile of 5-FUrd in proliferating human tissues remains to be fully characterized.

## Figures and Tables

**Table 1:** MIC values of antifungal drugs against reference *Candida* species. 5-FUrd, 5-fluorouridine; AMB, amphotericin B; FLU, fluconazole; VOR, voriconazole; CAS, caspofungin; 5-FC, flucytosine.

*Candida* Species	MIC (µg/mL)					
	5-FUrd	AMB	FLU	VOR	5-FC	CAS
*C. albicans* ATCC 10231	0.4	3.2	204.8	102.4	0.4	0.4
*C. auris* ATCC MYA-5001	409.6	409.6	1.6	0.4	1.6	0.8
*C. albicans* ATCC 90028	0.8	204.8	409.6	102.4	102.4	0.8
*C. albicans* ATCC 90029	204.8	204.8	409.6	409.6	204.8	6.4
*C. glabrata* ATCC 15126	12.8	12.8	6.4	0.05	0.02	0.05
*C. krusei* ATCC 14243	6.4	12.8	25.6	0.4	12.8	0.8
*C. parapsilosis* ATCC 22099	0.2	12.8	6.4	1.6	12.8	0.8
*C. tropicalis* ATCC 13803	51.2	6.4	12.6	0.4	0.05	0.05
*C. lusitaniae* ATCC 34449	51.2	12.8	1.6	0.02	0.05	0.4
*C. kefyr* ATCC 204093	51.2	51.2	0.1	0.8	0.4	0.8
*C. norvegensis* ATCC 22977	25.6	6.4	51.2	0.8	25.6	0.8

**Table 2:** In vitro antifungal interaction between 5-FUrd and other drugs against reference *Candida* species based on the checkerboard assay. FIC_A_, Fractional Inhibitory Concentration of 5-FUrd; FIC_B_, Fractional Inhibitory Concentration of the co-administered antifungal; FICI, Fractional Inhibitory Concentration Index.

*Candida* Species	Drug Combination	FIC_A_	FIC_B_	FICI	Interpretation
*C. albicans* ATCC 10231	5-FUrd + AMB 5-FUrd + FLU 5-FUrd + VOR 5-FUrd + 5-FC 5-FUrd + CAS	1 0.03 0.03 2 0.125	1 0.25 0.25 1 0.25	2 0.28 0.28 3 0.375	Indifference Synergism Synergism Indifference Synergism
*C. albicans* ATCC 90028	5-FUrd + AMB 5-FUrd + FLU 5-FUrd + VOR 5-FUrd + 5-FC 5-FUrd + CAS	1 0.25 0.125 0.5 0.125	1 0.015 0.062 1 0.5	2 0.265 0.187 1.5 0.65	Indifference Synergism Synergism Indifference Indifference
*C. glabrata* ATCC 15126	5-FUrd + AMB 5-FUrd + FLU 5-FUrd + VOR 5-FUrd + 5-FC 5-FUrd + CAS	0.5 0.5 1 0.25 0.125	0.5 1 1 0.5 1	1 1.5 2 0.75 1.125	Indifference Indifference Indifference Indifference Indifference
*C. krusei* ATCC 14243	5-FUrd + AMB 5-FUrd + FLU 5-FUrd + VOR 5-FUrd + 5-FC 5-FUrd + CAS	2 0.5 1 0.5 0.125	1 0.5 1 0.25 0.5	3 1 2 0.75 0.625	Indifference Indifference Indifference Indifference Indifference
*C. parapsilosis* ATCC 22099	5-FUrd +AMB 5-FUrd + FLU 5-FUrd + VOR 5-FUrd + 5-FC 5-FUrd + CAS	0.5 1 1 1 0.5	0.25 2 2 0.5 0.5	0.75 3 3 1.5 1	Indifference Indifference Indifference Indifference Indifference
*C. tropicalis* ATCC 13803	5-FUrd +AMB 5-FUrd + FLU 5-FUrd + VOR 5-FUrd + 5-FC 5-FUrd + CAS	1 0.5 1 0.25 0.031	1 2 1 1 0.5	2 2.5 2 1.25 0.531	Indifference Indifference Indifference Indifference Indifference
*C. lusitaniae* ATCC 34449	5-FUrd +AMB 5-FUrd + FLU 5-FUrd + VOR 5-FUrd + 5-FC 5-FUrd + CAS	1 1 1 1 1	1 2 2 2 1	2 3 3 3 2	Indifference Indifference Indifference Indifference Indifference
*C. kefyr* ATCC 204093	5-FUrd +AMB 5-FUrd + FLU 5-FUrd + VOR 5-FUrd + 5-FC 5-FUrd + CAS	1 1 1 0.125 0.031	0.5 3 1 0.5 0.1	1.5 4 2 0.625 0.131	Indifference Indifference Indifference Indifference Synergism
*C. norvegensis* ATCC 22977	5-FUrd +AMB 5-FUrd + FLU 5-FUrd + VOR 5-FUrd + 5-FC 5-FUrd + CAS	1 1 1 1 0.062	1 1 1 0.25 0.1	2 2 2 1.25 0.162	Indifference Indifference Indifference Indifference Synergism

**Table 3:** MIC values of antifungal drugs against clinical *C. albicans* isolates.

Number of Clinical *C. albicans* Strain	MIC (µg/mL)					
	5-FUrd	AMB	FLU	VOR	5-FC	CAS
1	0.1	1.6	409.6	204.8	0.1	0.8
2	0.1	25.6	409.6	204.8	0.4	0.4
3	0.1	12.8	3.2	102.4	0.1	0.2
4	0.2	51.2	409.6	204.8	0.2	0.2
5	0.4	12.8	204.8	409.6	0.8	0.8
6	0.4	25.6	409.6	204.8	0.4	0.2
7	0.4	25.6	409.6	204.8	0.4	0.4
8	0.4	51.2	204.8	204.8	0.4	0.4
9	0.4	1.6	409.6	204.8	0.8	1.6
10	12.8	1.6	0.8	0.1	0.1	0.4
11	1.6	51.2	409.6	51.2	1.6	3.2
12	12.8	12.8	3.2	0.1	0.1	0.8
13	0.1	25.6	409.6	204.8	0.4	0.4
14	0.2	25.6	409.6	204.8	0.4	0.4
15	0.8	12.8	409.6	204.8	0.4	0.8
16	0.4	25.6	409.6	204.8	0.4	0.8
17	12.8	25.6	12.5	12.5	0.4	1.6
18	1.6	12.8	6.4	12.8	0.8	0.2
19	1.6	12.8	12.8	102.4	0.4	0.2
20	0.1	51.2	409.6	204.8	0.4	0.8
21	0.8	51.2	409.6	204.8	0.8	0.8
22	0.8	51.2	409.6	409.6	0.8	0.8
23	12.8	12.8	1.6	0.4	0.1	0.1

**Table 4:** In vitro antifungal interaction between 5-FUrd and other drugs against clinical isolates of *C. albicans*, based on the checkerboard method. FIC_A_, Fractional Inhibitory Concentration of 5-FUrd; FIC_B_, Fractional Inhibitory Concentration of other antifungal drug; FICI, Fractional Inhibitory Concentration Index.

Number of Clinical *C. albicans* Strain	Drug Combination	FIC_A_	FIC_B_	FICI	Interpretation
1	5-FUrd + AMB 5-FUrd + FLU 5-FUrd + VOR 5-FUrd + 5-FC 5-FUrd + CAS	1 0.2 0.2 2 0.1	1 0.015 0.015 1 0.25	2 0.215 0.215 3 0.35	Indifference Synergism Synergism Indifference Synergism
2	5-FUrd + AMB 5-FUrd + FLU 5-FUrd + VOR 5-FUrd + 5-FC 5-FUrd + CAS	0.5 0.1 0.5 0.5 0.25	0.5 0.25 0.25 1 0.1	1 0.35 0.75 1.5 0.35	Indifference Synergism Indifference Indifference Synergism
3	5-FUrd + AMB 5-FUrd + FLU 5-FUrd + VOR 5-FUrd + 5-FC 5-FUrd + CAS	1 0.5 0.25 0.5 0.1	1 1 0.125 0.5 0.2	2 1.5 0.37 1 0.3	Indifference Indifference Synergism Indifference Synergism
4	5-FUrd + AMB 5-FUrd + FLU 5-FUrd + VOR 5-FUrd + 5-FC 5-FUrd + CAS	1 0.25 0.5 1 0.25	0.5 0.015 0.015 2 0.1	1.5 0.265 0.515 3 0.35	Indifference Synergism Indifference Indifference Synergism
5	5-FUrd + AMB 5-FUrd + FLU 5-FUrd + VOR 5-FUrd + 5-FC 5-FUrd + CAS	0.5 0.125 0.125 2 0.125	0.5 0.015 0.007 2 0.25	1 0.14 0.132 4 0.375	Indifference Synergism Synergism Indifference Synergism
6	5-FUrd + AMB 5-FUrd + FLU 5-FUrd + VOR 5-FUrd + 5-FC 5-FUrd + CAS	0.5 0.125 0.25 1 0.125	1 0.007 0.015 1 0.25	1.5 0.132 0.265 2 0.375	Indifference Synergism Synergism Indifference Synergism
7	5-FUrd + AMB 5-FUrd + FLU 5-FUrd + VOR 5-FUrd + 5-FC 5-FUrd + CAS	0.25 0.125 0.5 0.5 1	1 0.25 0.015 0.5 1	1.25 0.375 0.515 1 2	Indifference Synergism Indifference Indifference Indifference
8	5-FUrd + AMB 5-FUrd + FLU 5-FUrd + VOR 5-FUrd + 5-FC 5-FUrd + CAS	1 0.125 0.25 1 1	0.5 0.015 0.015 2 1	1.5 0.14 0.265 3 2	Indifference Synergism Synergism Indifference Indifference
9	5-FUrd + AMB 5-FUrd + FLU 5-FUrd + VOR 5-FUrd + 5-FC 5-FUrd + CAS	0.125 0.25 0.25 1 0.125	0.5 0.008 0.0156 2 0.25	0.625 0.258 0.265 3 0.375	Indifference Synergism Synergism Indifference Synergism
10	5-FUrd + AMB 5-FUrd + FLU 5-FUrd + VOR 5-FUrd + 5-FC 5-FUrd + CAS	0.5 1 1 0.0625 0.0156	0.0156 1 1 0.2 0.25	0.515 2 2 0.262 0.265	Indifference Indifference Indifference Synergism Synergism
11	5-FUrd + AMB 5-FUrd + FLU 5-FUrd + VOR 5-FUrd + 5-FC 5-FUrd + CAS	0.006 0.003 0.006 0.003 0.003	0.015 0.007 0.015 0.031 0.125	0.021 0.01 0.021 0.034 0.128	Synergism Synergism Synergism Synergism Synergism
12	5-FUrd + AMB 5-FUrd + FLU 5-FUrd + VOR 5-FUrd + 5-FC 5-FUrd + CAS	0.5 0.25 0.5 1 0.25	0.25 0.52 0.1 1 0.5	0.75 0.77 0.6 2 0.75	Indifference Indifference Indifference Indifference Indifference
13	5-FUrd + AMB 5-FUrd + FLU 5-FUrd + VOR 5-FUrd + 5-FC 5-FUrd + CAS	0.25 0.25 0.25 0.5 0.25	0.25 0.015 0.015 1 0.25	0.5 0.265 0.265 1.5 0.5	Synergism Synergism Synergism Indifference Synergism
14	5-FUrd + AMB 5-FUrd + FLU 5-FUrd + VOR 5-FUrd + 5-FC 5-FUrd + CAS	0.5 0.25 0.25 0.5 0.25	0.5 0.031 0.03 0.25 0.25	1 0.281 0.28 0.75 0.5	Indifference Synergism Synergism Indifference Synergism
15	5-FUrd + AMB 5-FUrd + FLU 5-FUrd + VOR 5-FUrd + 5-FC 5-FUrd + CAS	1 0.5 0.125 2 1	1 0.031 0.061 2 1	2 0.531 0.186 4 2	Indifference Indifference Synergism Indifference Indifference
16	5-FUrd + AMB 5-FUrd + FLU 5-FUrd + VOR 5-FUrd + 5-FC 5-FUrd + CAS	0.5 0.125 0.5 1 1	0.5 0.125 0.031 4 1	1 0.25 0.531 5 0.5	Indifference Synergism Indifference Antagonism Indifference
17	5-FUrd + AMB 5-FUrd + FLU 5-FUrd + VOR 5-FUrd + 5-FC 5-FUrd + CAS	0.004 0.004 0.004 0.5 1	0.25 0.25 0.25 2 1	0.254 0.254 0.254 2.5 2	Synergism Synergism Synergism Indifference Indifference
18	5-FUrd + AMB 5-FUrd + FLU 5-FUrd + VOR 5-FUrd + 5-FC 5-FUrd + CAS	1 0.25 0.25 0.5 0.25	2 0.125 0.031 0.25 0.1	3 0.375 0.281 0.75 0.31	Indifference Synergism Synergism Indifference Synergism
19	5-FUrd + AMB 5-FUrd + FLU 5-FUrd + VOR 5-FUrd + 5-FC 5-FUrd + CAS	1 0.25 0.25 1 1	1 0.25 0.062 2 1	2 0.5 0.312 3 2	Indifference Synergism Synergism Indifference Indifference
20	5-FUrd + AMB 5-FUrd + FLU 5-FUrd + VOR 5-FUrd + 5-FC 5-FUrd + CAS	0.25 0.12 0.05 1 0.5	0.031 0.015 0.015 4 0.5	0.281 0.135 0.065 5 1	Synergism Synergism Synergism Antagonism Indifference
21	5-FUrd + AMB 5-FUrd + FLU 5-FUrd + VOR 5-FUrd + 5-FC 5-FUrd + CAS	1 0.25 0.25 2 1	0.062 0.015 0.031 4 1	1.062 0.265 0.281 6 2	Indifference Synergism Synergism Antagonism Indifference
22	5-FUrd + AMB 5-FUrd + FLU 5-FUrd + VOR 5-FUrd + 5-FC 5-FUrd + CAS	0.25 0.062 0.062 1 0.25	0.25 0.015 0.015 1 0.25	0.5 0.077 0.077 2 0.5	Synergism Synergism Synergism Indifference Synergism
23	5-FUrd + AMB 5-FUrd + FLU 5-FUrd + VOR 5-FUrd + 5-FC 5-FUrd + CAS	1 0.5 0.5 0.5 1	1 0.5 0.5 0.5 1	2 1 1 1 2	Indifference Indifference Indifference Indifference Indifference

**Table 5:** MIC values of 5-FUrd in resistant *Candida* strains with and without EPIs. Arrows in parentheses indicate a decrease in MIC in the presence of EPI.

*Candida* Strain	MIC (µg/mL)					
	CCCP (µg/mL)			Verapamil (µg/mL)		
	0	5	10	0	5	10
*C. albicans* ATCC 90029	204.8	204.8	204.8	204.6	204.8	204.8
*C. auris* ATCC MYA-5001	409.6	409.6	409.6	409.8	409.6	409.6
*C. glabrata* ATCC 15126	12.8	12.8	12.8	12.8	12.8	12.8
*C. krusei* ATCC 14243	6.4	0.4 (↓)	0.1 (↓)	6.4	3.2 (↓)	1.6 (↓)
*C. tropicalis* ATCC 13803	51.2	51.2	51.2	51.2	51.2	51.2
*C. lusitaniae* ATCC 34449	51.2	51.2	25.6 (↓)	51.2	51.2	51.2
*C. kefyr* ATCC 204093	51.2	51.2	51.2	51.2	51.2	25.6 (↓)
*C. norvegensis* ATCC 22977	25.6	25.6	25.6	25.6	25.6	25.6
*C. albicans* clinical isolate no. 10	12.8	12.8	12.8	12.8	12.8	12.8
*C. albicans* clinical isolate no. 12	12.8	12.8	12.8	12.8	12.8	12.8
*C. albicans* clinical isolate no. 17	12.8	12.8	12.8	12.8	12.8	12.8
*C. albicans* clinical isolate no. 23	12.8	12.8	12.8	12.8	12.8	12.8

## Data Availability

The original contributions presented in this study are included in the article. Further inquiries can be directed to the corresponding author.
